# HMC-net: a ResNet fused hierarchical multi-scale cross-attention architecture for mammographic breast malignancy recognition incorporating explainable AI

**DOI:** 10.3389/fonc.2026.1787210

**Published:** 2026-04-13

**Authors:** Maria Fatima, Razia Zia, Irfan Ahmed Usmani, Dinara Turzhanova, Rahmat Ullah

**Affiliations:** 1 Department of Electronic Engineering, Sir Syed University of Engineering and Technology, Karachi, Pakistan; 2 Department of Computer Science, Faculty of Engineering Science and Technology, Iqra University, Karachi, Pakistan; 3 Biomedical Engineering Department, Salim Habib University (Formerly Barrett Hodgson University), Karachi, Pakistan; 4 Astana Medical University Scientific Research Institute of Radiology Named After Zhangali Khamzabayevich Khamzabayev Khamzabayev, Astana, Kazakhstan; 5 School of Computer Science and Electronic Engineering, University of Essex, Colchester, United Kingdom

**Keywords:** breast cancer diagnosis, deep learning, explainable AI, Grad-CAM, hierarchical self-attention, mammogram classification, multi-scale cross-attention, Resnet

## Abstract

Accurate and understandable interpretation of mammograms is fundamental to dependable identification of breast cancer, which facilitates clinical trust and usefulness. The framework proposed in this paper is known as the ResNet50HierarchicalMultiScaleCross-Attention (HMC) Network, building upon ResNet50 by embedding Hierarchical Self Attention and Multi-Scale Cross Attention modules for enriched feature representation toward mammogram-based detection of breast cancer. Intra-layer self-attention together with inter-layer cross-attention may enable the model to learn local as well as global patterns and hence improve performance for classification tasks on the MIAS dataset. For explainability issues, Grad-CAMs, Grad-CAM++, and Score-CAM are included in the architecture. Such methods yield heatmaps whose more clinically relevant regions can be made explicit for automated diagnostics to become transparent and trusted. With a 5-fold cross-validation run, it attained a mean accuracy of 0.9972 (± 0.05), a mean precision value of 0.9851 (± 0.13), a recall of 0.9899 (± 0.19), F1-score that amounted to 0.9864 (± 0.07). The values for AUC-ROC and specificity were found to be quite high at 0.99(± 0.01) and about 0.9978(± 0.09), respectively, basically beating most baseline models like ResNet50, VGG19, VGG16, and ViT among others in performance metrics variance as indicated by the Friedman test (p-value=0.002< 0.05). Between ResNet50 with Hierarchical Attention, ResNet50 with Multi-Scale Attention, and the proposed model using the Nemenyi *post-hoc* test, HMC-Attention clearly outperformed standard ResNet50; learning curves for stable convergence with limited overfitting provided evidence-based support that mammogram analysis is both accurate and transparent: the new baseline for automated diagnostics. This framework unites sturdy, profound implementation of deep learning with medical elucidation, setting paths toward trustworthy computer-supported diagnostic tools.

## 1 Introduction

Breast cancer is the most frequent malignancy among women, and it stays at the second spot in terms of oncology-related mortality within this group (1). Early detection of Breast Cancer (BC) significantly contributes to reducing mortality rates (2). Mammography remains the imaging technique most commonly employed to detect breast cancer at an initial phase in medical practice (3). The general perception about mammography is that it constitutes the most accurate screening process due to its low cost as well as its easy availability (4). Mammograms typically present certain radiological patterns that aid in the identification of breast neoplasms (5). Variability in the interpretation of radiological assessments exists among practitioners since it relies on individual judgment and expertise, which may lead to missed lesions and delayed diagnosis. Recent studies have emphasized the potential of newly developed imaging and hyperspectral CAD solutions for the diagnosis of breast cancer, while also pointing out the difficulties that still need to be overcome in the standardization of these solutions (6, 7). Even though CNNs have taken dominance in medical image analysis to extract fine spatial hierarchies, there exists a limitation for them to acquire global contextual information due to an inherently localized receptive field (8). Many recent architectures introduced customized components and enhancements to reinforce their predictive ability (9–17). The addition of extra layers is not sufficient in making a network accurate because it may encounter problems with learning due to gradient explosion as well as gradient decay (16). Different normalization methods have been introduced (18–21) to deal with these issues and enhance the training stability and convergence of deep neural networks.

When the depth of the network is increased, a phase is reached where the accuracy either plateaus or sometimes even decreases, thus indicating a diminishing return that cannot be explained by overfitting alone (11). The problem is overcome by He et al. with the idea of learning residual images, that is, learning a residual function with respect to an approximation, obtained in a previous layer, which leads to the design of layers that learn residual functions rather than mappings (11). Although highly efficient, the resulting ResNet models are huge, composed of 18 to 152 layers. In our research, we upgraded the ResNet architecture by integrating hierarchical self-attention and multi-scale cross-attention modules. In this, we obtained a reduced number of layers with high accuracy.

Attention mechanisms have taken strong research interest in recent times and have resulted in significant advances in the literature (21–24). Attention mechanisms were originally introduced in natural language processing tasks to emphasize the necessary linguistic components and suppress unnecessary details (25). The application of attention mechanisms to suppress noise was introduced by Hu et al. (22), which boosts the overall accuracy of models in classification tasks. Fu et al. (26) improved the state-of-the-art in semantic segmentation, with strong results on challenging datasets. In segmenting and classifying images, self-attention captures long-range dependencies by computing features (27). The use of hierarchical self-attention in capturing global and local features with efficiency and flexibility was shown by Yun Liu et al. (28). The mechanism is useful for learning local, context, and other features, with applications in different tasks (29–32).

Despite the success, self-attention has problems with increasing memory and computational requirements when handling large feature representations, giving rise to a number of approaches to reduce such requirements. Criss-cross attention (33) helped in relieving computational and memory requirements, while Li et al. (34) optimized self-attention via expectation-maximization clustering. The use of object-contextual representations in semantic segmentation was proposed by Yuan et al. (35), who concluded that label-guided context vectors are highly essential. A more efficient technique to define the overall context for semantic segmentation than matrix decomposition was proposed by Geng et al. (36). The employment of cross-transformers with two branches to identify small and huge structural patterns in multi-scale cross-transformers is illustrated by Torres et al. (37).

The self-attention mechanism used in the previous models compares keys and queries within the same feature map, but our mechanism combines hierarchical self-attention within individual layers and multi-scale cross-attention between layers. The use of two attention mechanisms enhances the capability of the model to identify the local and global information necessary when differentiating between a benign, malignant, and normal instance. Using end-to-end backpropagation, the proposed mechanism is optimized effectively, abolishing the need for iterative algorithms or additional semantic information. This effective method easily integrates with ResNet50, making it suitable for medical imaging since both accuracy and interpretability are vital.

However, the current architectures like RDTNet (38) and DBFA Net (39) depend on deformable transformers or dual branch architectures to model fine and global features. There is a significant gap in the literature to develop a unified and lightweight architecture that can model hierarchical and cross-scale features while maintaining interpretability. This paper fills this gap by incorporating hierarchical self-attention and multi-scale cross-attention mechanisms directly into a ResNet50 architecture to learn context-aware representations without using additional branches that can provide clinically interpretable explanations. Despite the strong validation and test performances of the DL models, they might miss the patterns aligning with human expertise or domain understanding (40, 41). Explainable AI techniques provide human-understandable explanations, which depict how decisions are reached by black-box models (40–42). The role of attention mechanisms and their transparency as black-box components have not been investigated in depth. This paper investigates this gap by studying the performance of attention mechanisms in classification tasks using explainable AI (XAI) methods.

Following a quantitative and qualitative approach, our evaluation first focuses on the quantitative performance metrics of accuracy, precision, and F1-score for CNNs with and without AMs. Qualitatively, an analysis of the effects introduced by AMs is done using XAI. Based on this, we identify the following key contributions:

1. A hybrid architecture embedding hierarchical self-attention and multi-scale cross-attention within ResNet50, capable of modeling both local and global feature interactions.
2. Integrating three complementary XAI visualization techniques for better diagnostic transparency.
3. Strict validation for the MIAS database using five-fold cross-validation with statistical testing, Friedman + Nemenyi.
4. Demonstration of how HMC-Net improves the diagnostic accuracy and explainability by overcoming major trade-offs in automatic screening for breast cancer.

The rest of the study is organized as: Background and literature review are presented in Section 2, while the methodology adopted in this study is presented in Section 3. Section 4 presents experimental results with interpretation and statistical analysis. Finally, the conclusion is summarized in the last section.

## 2 Related works

Cancerous lesion identification in breast mammograms remains one of the prominent areas of study in both recent and ongoing efforts in research. Deep learning approaches have drastically improved the field of medical image analysis, especially in the classification of mammograms. While CNNs achieve strong results, they often struggle to capture both fine local details and broader contextual patterns. Attention mechanisms have been introduced to address these gaps, improving feature representation and interpretability, though most existing methods still lack effective multi-scale integration tailored to mammographic structures. In response to these challenges, our work introduces a novel fused Attention-Driven Deep Dense Network tailored for breast cancer classification, rigorously evaluated on the well-established MIAS dataset. Only a limited number of studies have incorporated statistical testing to evaluate the significance of architectural modifications and their combined effect on overall model performance.

### 2.1 Traditional machine learning and standard CNN-based classification

Numerous studies have examined various machine learning approaches for classifying breast cancer. M. M. Alshammari et al. (43)proposed a CAD system, powered by machine learning, constructed through a series of image processing steps organized into multiple stages. The findings recommend using an optimized Support Vector Machine or Naïve Bayes classifier (44). The study identified GLCM (Gray Level Co-Occurrence Matrix) with Random Forest as the optimal feature-classifier pair in the first stage, achieving 97% accuracy with F1-scores of 0.98 (normal) and 0.97 (abnormal) (45). MLP, KNN, GP, and RF were applied to the WBCD dataset, with RF achieving the best performance at 96.24% accuracy (46).The study proposed a framework using five supervised ML models for classification. In (47), the author employed six machine learning models for breast cancer diagnosis. Liu et al. (48) proposed an adaptive wavelet thresholding method that embeds threshold selection within deep learning by treating thresholds as trainable parameters in the CNN framework. Islam et al. (49) presented a CNN-based model for classifying IDC breast cancer, incorporating nine convolutional layers, three max-pooling layers, four dropout layers, and two fully connected layers. The model achieved an accuracy of 89% in IDC detection.

### 2.2 CNN architectures utilizing transfer learning

Transfer learning (TL), also referred to as pre-trained learning, has been extensively applied in the medical field alongside deep learning and has been demonstrated in several earlier studies (25–27) to outperform conventional approaches. R. Mehra et al. (50) assessed ResNet-50, VGG-16, and VGG-19 for feature extraction, and the extracted features were subsequently used to train a logistic regression (LR) classifier. A. M. Zaalouk et al. (51) examined five pre-trained CNN models for binary and multi-class classification using a two-stage training approach: first, they froze all layers except the fully connected ones. Then they did fine-tune. H. Mewada et al. (52) adopted the DenseNet161 framework, coupling a dynamic residual block to improve feature learning. V. Kumari et al. (53) adopted Xception, VGG-16, and DenseNet-201 models for image classification of breast cancer. In (54), MVGG, which is an altered variant of VGG, was coupled with a mobile network for breast cancer diagnosis employing the DDSM mammography image database. L. G. Falconi et al. (55) developed a multi-model concept that consisted of VGG, ResNet, ResNext, and Xception models. In research work (56), digital mammograms were processed for analysis employing the Transfer Learning (TL) technique.

### 2.3 Neural models based on transformer architecture

Most recently, it has been observed that transformer-based models turn out to be quite effective in computer vision because of their inherent strength in modeling long-range dependencies and contextual relationships (57). Their work proposed a novel hybrid deep dense learning framework by combining deep transfer learning and a transformer. The method focuses on key mammographic features, extracts high-value paired information with the help of the ViT-L16 transformer, and assures an accuracy of 98.08%. S. Tummala et al. (58) used the applicability of Swin Transformers. The findings of their study indicated that the performance of the ensemble model achieved an accuracy of 93.4%. R. M. Al-Tam et al. (59) introduced a hybrid multi-class classification technique on pathological images by integrating depthwise separable convolutional neural networks with transformers for utilizing local and global features accordingly. There exist some works that investigate the adoption of Transformer architectures over the ultrasound images in the task of classifying breast lesions (60, 61) (62). The author presented a method that utilized a CNN module for breast ultrasound images to capture local features and incorporated a ViT module to model global relationships between different regions while enhancing relevant local features. The CNN component, referred to as the VGGA module, consisted of a VGG backbone, a fully connected feature extraction layer, and a squeeze-and-excitation block. Additionally, several studies have utilized ViT architectures pre-trained on large-scale datasets for the classification of skin cancer (63, 64).

### 2.4 Hybrid CNN models with XAI

(65)developed an XAI-based deep learning model with enhanced DenseNet architecture and fine-tuning (42).This research utilized patient diagnostic data, along with various machine learning classifiers, to detect breast cancer. The importance of using interpretability techniques like SHapley Additive exPlanations (SHAP), Local Interpretable Model-agnostic ExPlanations (LIME), Explain Like I’m Five (ELI5), Anchor, and Quantum Lattice (QLattice) was emphasized in the study for a clearer understanding of the results of the model (66). The new CatBoost+MLP model developed was validated using Shapley Additive Explanation values. to obtain the significance of the features. **Table 1** shows a comparison of these contributions.

**Table 1:** Summary of literature on breast cancer classification using ML, deep learning, transfer learning, hybrid deep learning-transformer models, and XAI.

Reference	Year	Study domain	Dataset used	Model for classification	Description
M. M. Alshammari et al. (43)	2021	Diagnosis of Breast Cancer	Mammograms	KNN, SVM Decision Tree, Naive Bayes, Discriminant Analysis	The study presents an ML-based CAD system, recommending the use of an optimized Support Vector Machine or Naïve Bayes classifier for optimal results.
M. S. Darweeshet al. (44)	2021	Diagnosis of Breast Cancer	Mammograms	Random Forest	The study employed GLCM and LBP, identifying Random Forest as the best classifier for both stages.
A. Bhardwajet al. (45)	2022	Breast Cancer Classification	Wisconsin Breast Cancer Database	Random Forest, KNN, MLP, GP	The study applied MLP, KNN, GP, and RF to the WBCD dataset, identifying RF as the top-performing classifier.
A. Kumar et al. (46)	2024	Breast Cancer Detection and Identification	Wisconsin Breast Cancer dataset.	DT, RF, SVM, XGBoost, and ANN	It proposed a framework using five Machine learning algorithms, where RF and XGBoost performed with similar accuracy, SVM achieved the best ROC performance along with the shortest training time, and ANN had the longest training duration.
A. Khalid et al. (47)	2023	Breast Cancer Detection and Prevention	Wisconsin Breast Cancer dataset.	Random Forest, Decision Tree, KNN, Logistic Regression, Support Vector Classifier, and Linear Support Vector	The models were constructed using a wide array of machine learning algorithms.
Y. Liu et al. (48)	2024	Breast cancer classification	histopathological image BreakHis, BACH	CNN model	This study proposed a thresholding approach that incorporates the selection of thresholds within deep learning in a way that the threshold is treated as a trainable parameter within the CNN model.
T. Islam et al. (49)	2024	Classification of breast carcinoma	histopathological image	CNN model	This work presented a CNN-based framework developed for the identification of IDC and metastatic breast cancer.
A. M. Zaalouk et al. (51)	2022	Breast Cancer Diagnosis	BreakHis	VGG19, ResNet152, Inception, ResNetV2, DenseNet-201, Xception	Five pre-trained CNN models were evaluated using data augmentation, and a transfer learning approach was proposed.
V. Kumari et al. (53)	2023	Breast Cancer Classification	Invasive Ductal Carcinoma (IDC) dataset and BreaKHis	VGG16, Xception, and Densenet-201	This research presented a transfer learning-based system designed to identify breast cancer using histopathological images of breast tissue.
A. Khamparia et al. (54)	2021	Diagnosis of breast cancer	MammogramDDSM	Modified VGG	The premise of this paper is to utilize transfer learning through the application of a modified VGG, applied to 2D and 3D mammogram image data.
S. U. R. Khan et al. (57)	2024	Breast Cancer Tumor Detection	INbreast	ResNet50, EfficientNetB1, and the proposed ProDense	By combining the ViT-L16 transformer with CNN, the approach targets critical features in mammography
S. Tummalaet al. (58)	2022	Breast Cancer Classification	BreaKHis	ensemble model	This study examined the effectiveness of an ensemble approach using Swin Transformers.
X. Qu et al. (62)	2022	Breast Cancer classification	Breast Ultrasound	VGGA	The proposed approach uses a vision transformer (ViT) for global relationships and a CNN for local feature extraction.
M. A. Talukder et al. (65)	2025	Breast cancer detection	BreakHis BACH	DenseNet	This research presented an XAI-based deep learning model for breast cancer detection, featuring tailored DenseNet modifications using BN-ReLU-Conv and Block-End layers, combined with optimized fine-tuning.
T. Arravalli et al. (42)	2025	Breast cancer detection	UCTH Breast Cancer Dataset	Random forest, KNN, Logistic regression, Adaboost, Decision Tree, Catboost, Lightgbm, Xgboost, STACK	This research utilized patient diagnostic data alongside various machine learning classifiers to detect breast cancer and incorporated explainable AI to improve model transparency.
P. N. Srinivasu et al. (66)	2024	Analyzing breast cancer	Breast Cancer Wisconsin	CatBoost algorithm with a multi-layer perceptron neural network	The CatBoost+MLP model was analyzed using Shapley values to assess feature importance.

Moreover, current approaches tend to emphasize either local or global information, causing a gap in dealing with multi-scale patterns that play a significant role in effective mammographic image classification. This highlights the importance of models with emphasis on both hierarchical and cross-scale attentions, which would add more information to the features by allowing the detection of subtle lesions through their attention to both fine and global aspects.

## 3 Proposed methodology and materials

**Figure 1:**
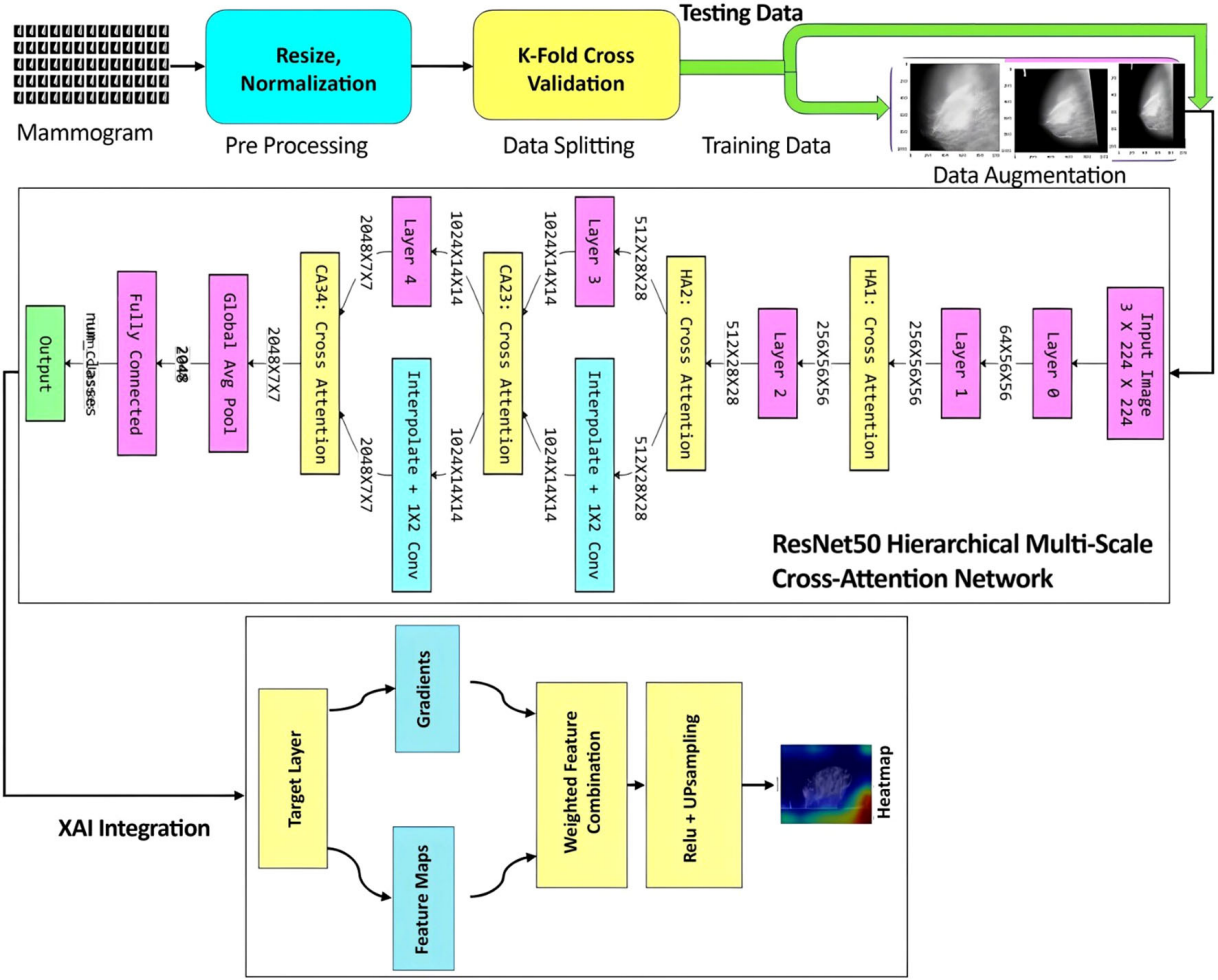
A block diagram of the proposed framework.

This section describes the methodology that is used for the research work. It describes the data set, preprocessing approaches, splitting of data, data augmentation, and the proposed architecture for the research work. A block diagram of the proposed framework is shown in **Figure 1**.

At the core of this framework is the deployment of an attention mechanism with ResNet50, which aims to harness both global and local feature representations. Firstly, the deployed attention mechanism is responsible for learning global context information over the entire input, thereby making it apt for capturing global context. Conversely, the existing ResNet50 network is proficient in learning local features by concentrating on certain spots in the data. Therefore, by integrating both these approaches, it is possible to harness their complementary functionalities. These include harnessing global context information through the attention mechanism, apart from improving local feature extraction using ResNet50. Both of these approaches tend to work in tandem by individually overcoming the existing drawbacks of traditional convolutional neural networks that tend to learn only local features. Therefore, by integrating both approaches, it is possible to harness a more robust feature representation that comprises both global context information as well as local features extracted from the data.

### 3.1 Dataset and preprocessing

**Figure 2:**
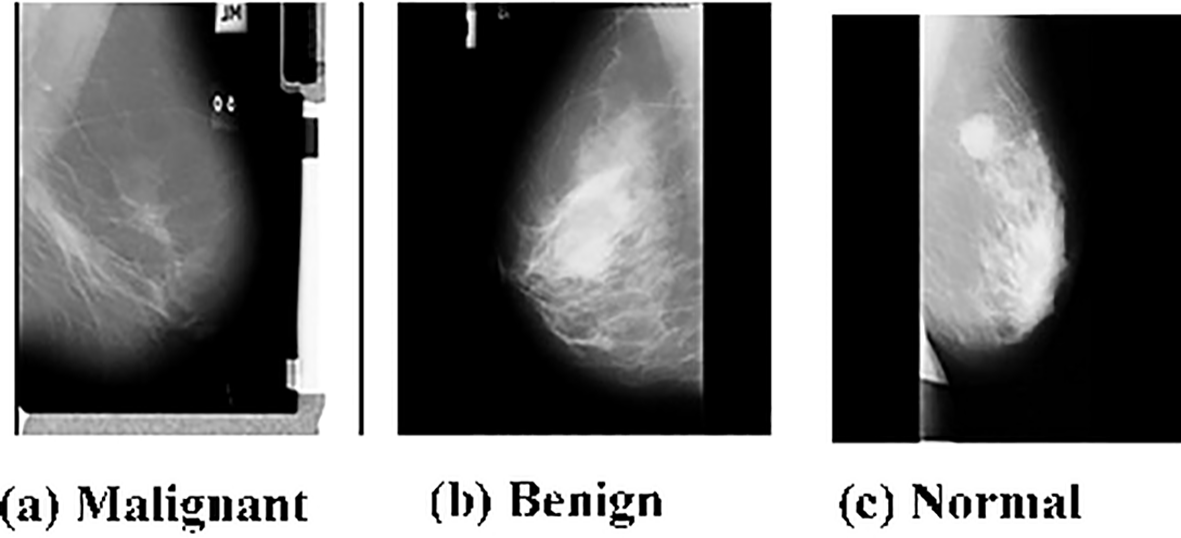
Representative samples from the dataset.

The dataset utilized in this research is the publicly accessible MIAS dataset, proposed by Suckling et al. (1994) (67). It contains 322 mammograms of 161 patients. Some of the samples from the data set MIAS are displayed in **Figure 2**.

In the training step, mammographic images are then acquiesced to a series of transformations that aim to increase the diversity of the data. Moreover, it helps in removing the problem of overfitting. This is achieved through image resizing to a fixed resolution of 224x224 pixels (68), typically suited to the input image specifications of commonly used convolutional neural networks, like those that were pretrained using a large number of images, like ImageNet (ResNet-50).

As the MIAS image database contains grayscale mammograms, it is transformed into a three-channel image by replicating the same image into the RGB channels for consistency with the CNN model accepting images with RGB values (8).Finally, to introduce variability related to image acquisition in a clinical setting, brightness and contrast adjustments of ±20% are carried out using color jittering on the replicated images.

The pixel intensities are normalized with a fixed mean and variance of 0.5 on each of the three channels. This step of normalizing the pixel values helps convert the input distribution into the range of [-1, 1], ensuring easier convergence of training since it is more numerically stable (8, 69).

### 3.2 Cross-validation

Cross-validation is a technique used for assessing the performance and generalizability of a model. It divides the dataset into numerous subsets/folds, which are then used for training and testing (70). The method adopted in this paper is known as k-fold cross-validation. Where k=5 (71), it implies that the dataset is partitioned into 5 equal/near-equal folds, where each fold is used as the test set once, while the remaining k-1, i.e., 4 folds, are used for training. This is also cycled through five times, ensuring that each data sample gets utilized for both training purposes as well as testing purposes. It helps reduce the issue of “Overfitting Bias”. Cross-validation helps remove the chances of being overfit to train-test splits. It helps generate “generalizability”. It helps make tests more robust by offering better predictions of the influence of models on test data, such as mammograms of new patients. It also helps with “data efficiency”. It maximizes the utilization of limited data in applications such as mammograms, which may have limited data samples.

### 3.3 Data augmentation

Quality and quantity of data are critical to deep neural networks, and thus, extensive datasets are vital for perfect training and good performance. Data augmentation is a regularization technique that helps enhance a model’s robustness and generalization (72). These are techniques for artificially increasing the size of an existing training dataset by creating modified versions of each sample. It helps in increasing the diversity in the data while avoiding overfitting during the learning process. For artificial expansion of the training dataset, to improve the generalizability, 200,000 synthetic image variants are generated using an augmentation strategy. Data augmentation was performed only on the training folds, and the test folds were left unchanged. Patient-level splitting was ensured before data augmentation, and the 5-fold cross-validation was patient-independent, meaning that all images of a patient were restricted to only one fold. This process eliminates data leakage.

The augmentation techniques applied in our study include purely geometric transformations (72) on the original images, and include:

#### 3.3.1 Random horizontal flipping

This operation is applied with a probability of 50%; it simulates the lateral variations in breast positioning and reinforces the capability of the model to recognize features independent of orientation (72).

#### 3.3.2 Random rotation

Within a range of ±15 degrees, images are randomly rotated to allow for minor misalignments during image acquisition, as it further reduces the sensitivity of the model to angular shifts (72).

**Figure 3:**
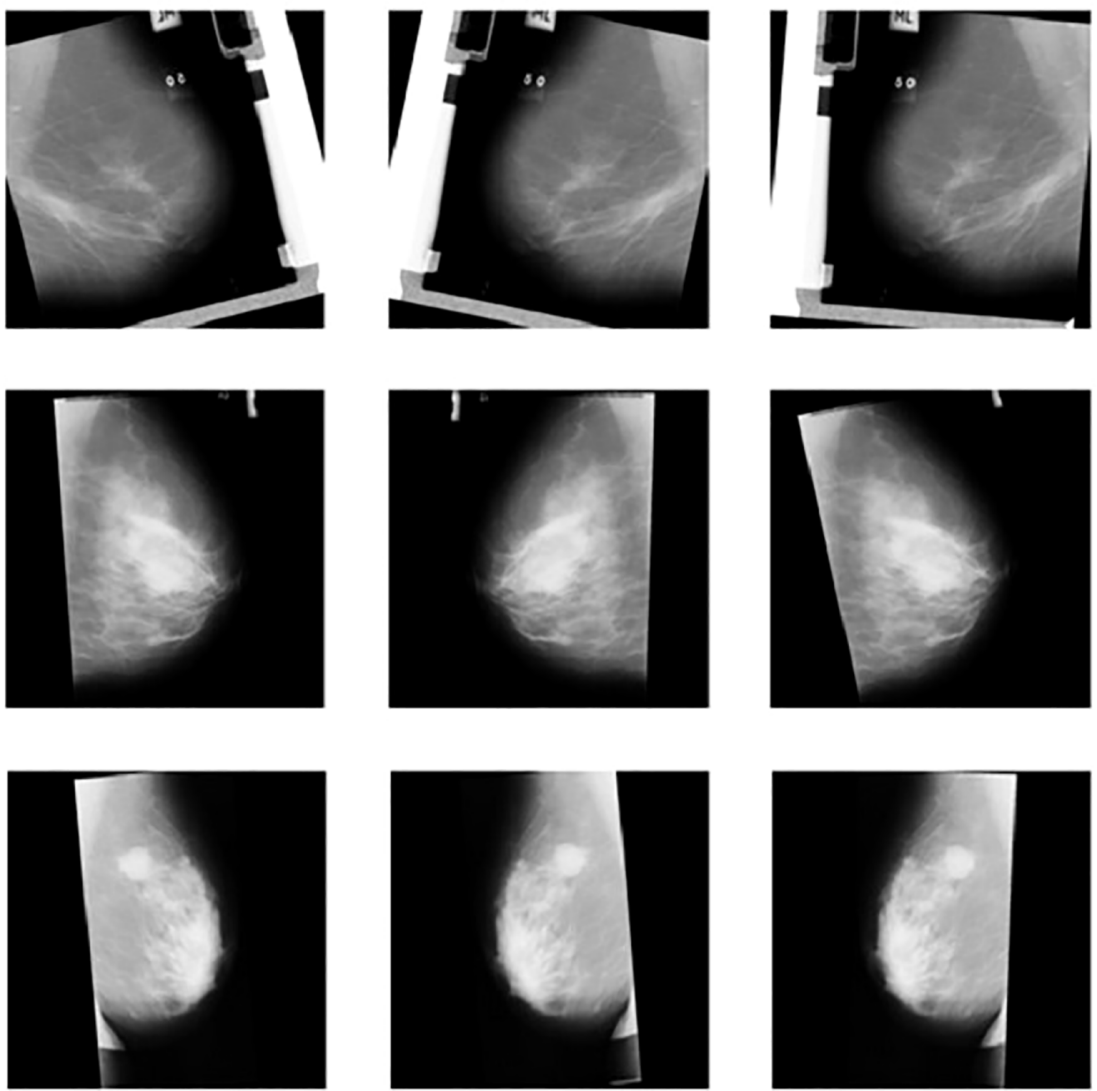
Sample images of mammograms after data preprocessing and augmentation.

These image transformations did not alter the key visual characteristics of the original images and mainly served to increase the training time of the model. **Figure 3** shows preprocessed mammogram images-resized, normalized, and augmented-ready for model training.

### 3.4 Proposed architecture

In this work, a new architecture is introduced by integrating the effectiveness of both the attention mechanism and the ResNet50 models in a perfect way, as shown in **Figure 1**. The proposed Hierarchical Multi-Scale Cross-Attention Mechanism is incorporated into a ResNet50 model to form a new model, called ResNet50HierarchicalMultiScale. Here, this proposed model nicely incorporates all standard ResNet50 convolutional layers with filter sizes 64, 256, 512, 1024, and a final filter size of 2048 for layers 0 through 4, respectively. Hierarchical self-attention modules, HA1 and HA2, are inserted after layers 1 and 2 with 256 channels (feature size 56x56) and 512 (feature size 28x28), respectively, in order to focus on local details such as microcalcifications and lesion edges. Additionally, multi-scale cross-attention modules (CA23, CA34) are incorporated between layer 2 and layer 3 with 1024 channels (feature size 14 × 14) and between layer 3 and layer 4 with 2048 channels (feature size 7 × 7) to facilitate the integration of low-level and high-level features across different spatial scales. Our model does not depend on pre-trained layers, unlike traditional architectures of attention-based augmentation, so the model can learn domain-specific features from scratch for medical imaging tasks. This new incorporation enhances the model’s ability to capture both localized and global contextual information, making it very powerful in distinguishing benign, malignant, and normal mammograms. Also, it is compatible with explainable AI techniques such as Grad-CAM, Grad-CAM++, and Score-CAM that could be potentially integrated with the current work for an improved interpretation.

**Figure 4:**
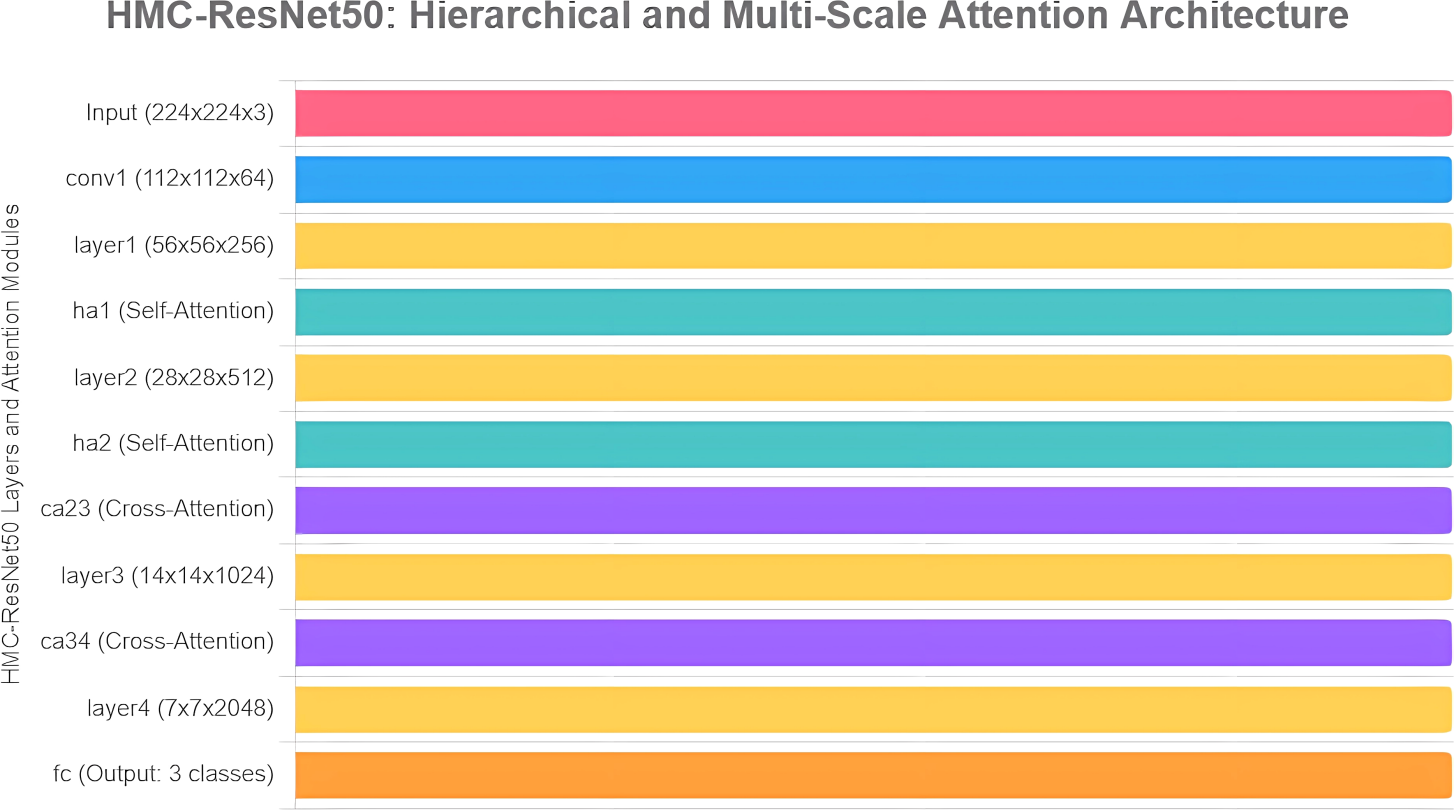
HMC-ResNet50 architecture in a block-style representation using Chart.js.

**Figure 4** depicts the HMC-ResNet50 architecture in a block-style representation, using Chart.js, where each block represents a ResNet50 layer or attention module along with its output dimensions. Hierarchical self-attention modules (HA1 and HA2) are placed after layer 1 and layer 2 to enhance local features, respectively, to provide precise localization in the heatmap. Cross-attention modules (CA23 and CA34) link layer2–layer3 and layer3–layer4, respectively, to merge information from different scales, which provides much better accuracy, 0.9972, and robustness.

#### 3.4.1 ResNet50 backbone

ResNet50 is a deep convolutional neural network with 50 layers, which aims to resolve the vanishing gradient problem using residual learning. The idea of ResNet50 was first introduced in 2015 by (73). Generally, a Residual Network adheres to two principles (1): The size of the output feature maps determines the number of filters in the following layer, and (2) when changing dimensions by half, doubling the output dimension will keep the complexity of computation equal in all layers (57). Our network utilizes ResNet50 layers (from Layer0 to Layer4) to capture increasingly higher-level abstractions with different numbers of channels. Every layer in our network contains residual connections to counter vanishing gradients. ResNet50 layers include Initial layers: a 7x7 convolutional layers named conv1, batch normalization layers named bn1, ReLU, Max pooling layers, Residual layers: layers 1 to 4 with increasing dimensions of channels from 256 to 256, 512 to 512, 1024 to 1024, and 2048 to 2048, with a reduced image dimension due to each stage’s striding nature, Final layers: an adaptive average pooling convolutional layers, a Fully connected layers for a class output. The parameter mappings in each layer of ResNet50 are given by:

x0: Output of layer 0 (initial conv, batch norm, ReLU, maxpool).
x1: Output of layer 1 (256 channels).
x2: Output of layer 2 (512 channels).
x3: Output of layer 3 (1024 channels).
x4: Output of layer 4 (2048 channels).

Let x ∈ *ℝ ^B×3×,H×W^* be the input image tensor, where B denotes the batch size, 3 is the number of color channels, and H×W is the spatial resolution.

Layer 0 (Initial Convolution, BatchNorm, ReLU, MaxPool), as shown in Equation 1:

(1) x0=MaxPool (ReLU(BN(Conv1(x))))

where Conv1 is a 7×7 convolution with 64 filters, stride 2, followed by batch normalization (BN), ReLU activation, and 3×3 max pooling with stride 2.

#### 3.4.2 Cross-attention mechanism

The Cross-Attention module draws inspiration from the attention mechanism of transformers. This module lets the model selectively focus on informative parts of the feature maps by calculating the relationships between a query feature map x and a key/value feature map y. This method allows the network to capture complex interdependencies and highlight significant regions within feature maps (74).

##### 3.4.2.1 Cross-attention block equations

The Cross-Attention module computes attention between a query feature map *X* and a key/value feature map *Y*, both through shape (*B*, *C*, *H*, *W*), where *B* is the batch size, *C* is the number of channels, and *H* × *W* is the spatial dimension.

In the ResNet50-Hierarchical Multi-Scale model, queries are obtained through a 1×1 convolution applied to specific layer feature maps (e.g., layer 1 for ha1, layer 4 for ca34) as expressed in Equation 2.

(2) Q = Wq * X    

where *Wq ∈ ℝ^C×C/8^* is a 1x1 convolution, and *Q ∈ ℝ^B,C/8,H,W^*.

In hierarchical self-attention (HA1, HA2), keys come from the same feature map as queries, capturing local relations. In multi-scale cross-attention (CA23, CA34), keys are taken from lower-level feature maps, resized and channel-aligned to *ℝ^B,1024,14,14^* as defined in Equation 3.

(3) K = Wk * Y   

where *W_k_* ∈ *ℝ^C×C/8^* and *K ∈ ℝ^B,C/8,H,W^*.

In hierarchical self-attention, values originate from the same feature map as queries and keys (e.g., X1​ for ha1), preserving feature details. In multi-scale cross-attention, values are taken from lower-level layers (e.g., X2′′ for CA23), enabling integration of fine-grained features into higher-level representations as expressed in Equation 4.

(4) V = Wv * Y   

where $Wv\;\in {\mathbb{R}}^{CxC,}$ and $V\;\in {\mathbb{R}}^{B,C,H,W\;V}$.

Here, ∗ denotes the convolution operation.

The feature maps are flattened spatially:


$$Q\to Q^{\prime }\in {\mathbb{R}}^{B,C/8,H\cdot W\;}$$

$$K\to K^{\prime }\;\in {\mathbb{R}}^{B,C/8,H\cdot W\;\;}$$

$$V\to V'\in {\mathbb{R}}^{B,C,H\cdot W\;}$$

The attention computation in our Hierarchical Multi-Scale Cross-Attention Mechanism, integrated into the ResNet50MultiScaleHierarchical model, is designed to enhance feature representation by capturing both local and cross-layer dependencies for mammogram classification.

Energy (Similarity) is defined in Equation 5, and Attention Weights are defined in Equation 6:

(5) $$E\;={\left(Q'\right)}^{T}K'\in {\mathbb{R}}^{B,H\cdot W,H\cdot W\;}$$

where (*Q*′)^T^ ∈ *ℝ ^B,H·W,C/8^* and · is matrix multiplication.

Attention Weights:

(6) $$A\;=\text{ softmax }\left(E,\text{ dim }=-1\right)\;\in {\mathbb{R}}^{B,H\cdot W,H\cdot W\;}$$

where softmax (*E_b,i,j_*)= $\frac{exp(\;E_{b,i,j})}{\sum_{j\;exp}(E_{b,i,j)}}$In the ResNet50-MultiScaleHierarchical model, the output of the cross-attention mechanism produces weighted feature representations, which are then integrated into the ResNet50 framework for mammogram classification. Weighted Values, Reshape, and residuals are defined in Equations 7–9.

Weighted Values:

(7) $$O'=V'\cdot \;A^{T}\;\in {\mathbb{R}}^{B,C,H\cdot W}$$

where *A*^T^ ∈ *ℝ^B,H·W,H·W^*.

Reshape and Residual:

(8) Reshape  O' to O ∈ ℝB,C,H,W

then:

(9) Out = γ · O + X 

where γ ∈ ℝ is a learnable scalar parameter initialized to 0.

#### 3.4.3 Hierarchical attention

Hierarchical attention applies attention on the same feature map at different ResNet50 layers (layer1 and layer2), refining each layer’s output by emphasizing important spatial regions. This is done using the HA1 and HA2 modules, which process the 256- and 512-channel feature maps from layer1 and layer2, respectively. Functionally similar to self-attention, both queries and keys/values come from the same feature map, enabling the model to better capture spatial dependencies within a layer (28).

In this case, the same feature map is utilized for query and key/value (self-attention) with equations shown above.

Layer 1 (ResNet Block with Hierarchical Attention) is expressed in Equation 10:

(10) x1=Layer1(x0) , x1'=HA1(x1,x1) 

where Layer1 is the first ResNet block outputting 256 channels, and HA_1_​ is the hierarchical cross-attention as shown in Equation 11:

(11) $$\text{HA}1\left(\text{x},\text{y}\right)=\text{γ}\cdot \text{Softmax}(\frac{\text{Q}\left(\text{x}\right)\text{K}\left(\text{y}\right)\text{T}}{\sqrt{d_{k}}}\text{V}\left(\text{y}\right)+\text{x}$$

with Q(x)=Conv_1×1_(x), K(y)=Conv_1×1_(y), V(y)=Conv_1×1_(y), and γ is a learnable scalar.

Layer 2 (ResNet Block with Hierarchical Attention) is expressed in Equation 12.

(12) x2=Layer2(x1'), x2'=HA2(x2, x2)   

Since Layer2 outputs 512 channels, HA_2_ is defined similarly to HA_1_.

#### 3.4.4 Multi-scale attention

The multi-scale attention mechanism allows the model to fuse information (37) from different layers of ResNet50, the convolutional backbone network, with each layer capturing information at progressively different spatial scales and semantic layers. Cross-scale attention is implemented between layer 2 (512 channels) and layer 3 (1024 channels) with ca23, and between layer 3 (1024 channels) and layer 4 (2048 channels) with ca34. To match their dimensions, 1x1 convolution (match_CA23 and match_CA34) is applied to match the channel dimensions of low-resolution feature layers with those of high-resolution layers. And, for spatial dimensions, sizes are normalized by F. interpolate to resize low-resolution feature layers to match those of high-resolution feature layers. This allows for better fusion of low-level fine features like edges and textures with high-level abstract concepts, ultimately optimizing performance on complex visual tasks such as mammogram analysis.

Layer 3 (ResNet Block with Multi-Scale Attention) is expressed in Equation 13:

(13) $${\text{x}}_{3}=\text{Laye}{\text{r}}_{3}\left({\text{x}}_{2}\prime \right),\;\;\;\;\;\;\;{\text{x}}_{2}\prime \prime =\text{Interpolate}\left(\text{Con}{\text{v}}_{1\times 1}\left({\text{x}}_{2}\prime ,512\to 1024\right)\right),\;\;\;\;\;\;{\text{x}}_{3}\prime =\text{C}{\text{A}}_{23}\left({\text{x}}_{3},{\text{x}}_{2}\prime \prime \right)$$

where Layer_3_ outputs 1024 channels, Conv1×1 match channel dimension, interpolate resizes x_2_′ to match x_3_’s spatial dimensions, and CA_23_ is the cross-attention mechanism,as shown in Equation 14:

(14) $$\text{C}{\text{A}}_{23}\left(\text{x},\text{y}\right)=\text{γ}\cdot \text{Softmax }\frac{\text{Q}\left(\text{x}\right)\text{K}\left(\text{y}\right)\text{T}}{\sqrt{d_{k}}}\;\text{ V}\left(\text{y}\right)+\text{x }$$

Layer 4 (ResNet Block with Multi-Scale Attention) is expressed in Equation 15:

(15) $${\text{x}}_{4}=\text{Laye}{\text{r}}_{4}\left(x_{3}\prime \right),\;\;\;\;x_{3}\prime \prime =\text{Interpolate}\left(\text{Con}{\text{v}}_{1\times 1}\left({\text{x}}_{3}\prime ,1024\to 2048\right)\right),\;\;\;{\text{x}}_{4}\prime =\text{C}{\text{A}}_{34}\left({\text{x}}_{4},{\text{x}}_{3}\prime \prime \right)$$

where Layer_4_ outputs 2048 channels, and CA_34_ is defined likewise.

#### 3.4.5 Classification

After inception modules with multiple branches, the final feature map is average-pooled and fed into a fully connected layer to obtain class logits, as shown in Equation 16.

(16) $$\text{z }=\text{ FC }\left(\text{Flatten }\left(\text{AvgPool }\left({\text{x}}_{4}\prime \right)\right)\right)$$

where AvgPool is adaptive average pooling to 1×1, Flatten converts to a vector. A fully connected layer FC mapping is used for output layer mapping, resulting in C = 3 classes.

### 3.5 Explainable artificial intelligence

Clarifications are useful in the verification of decisions made by AI, making the issue of interpretability an important consideration in the field of machine learning. While deep neural networks (DNNs) perform well from an accuracy perspective in numerous fields of application (73), the high number of parameters involved in the networks raises the issue of complexity, making them difficult to understand. High-performing metrics don’t necessarily imply conformity with human logic, meaning that the workings of the network’s prediction are not well understood until the underlying workings and logic of the network are fully grasped. This is, however, the challenge that is sought to be solved by the field of Explainable Artificial Intelligence (Explainable AI-XAI) (40).

#### 3.5.1 GRAD-CAM (gradient-weighted class activation mapping)

Grad-CAM (Gradient-weighted Class Activation Mapping) is an XAI method that maps the image areas that contributed the most to the prediction of the model (75). This is done by obtaining the feature maps from a selected convolutional layer (layer 4, for instance) during the forward phase and storing the gradients of the target class score with respect to the maps during the back-propagation phase. The gradients are averaged spatially, and this is used along with the maps. An application of the ReLU activation function follows, and the output is enlarged to the size of the image (224×224), making visible and important areas, for instance, in mammographic images, which contributed to the decision of the network, thus improving interpretability.

Grad-CAM generates a heatmap highlighting regions contributing to the prediction for a target class. Let A ∈ ***ℝ*** *^B,C,H,W^* be the feature maps from the target layer (layer 4), and *y*_c_​ be the score for class *c*.

##### 3.5.1.1 Gradients

Compute gradients of the class score with respect to the feature maps, as defined in Equation 17:

(17) $$G=\frac{\partial y_{c}}{\partial A}\;\;\in {\mathbb{R}}^{B,C,H,W}$$

where A∈ ℝ ^B^×^2048^×^H4^×^W4^ are the feature maps from Layer 4.

##### 3.5.1.2 Weights

Average the gradients spatially to get the importance of each feature map. The weight for the k-th channel is defined in Equation 18:

(18) $$\alpha_{k}=\frac{1}{H.W}\;\sum_{i=1}^{H}\sum_{j=1}^{W}G_{k,i,j}\in {\mathbb{R}}^{C}$$

where α*_k_* is the weight for the k-th channel, and ***k*** indexes the feature map channel.

##### 3.5.1.3 CAM (heatmap) computation

Combine the feature maps with their weights to obtain the class activation map (CAM) as defined in Equation 19:

(19) $$\text{CAM}=\text{ReLU}(\sum_{k=1}^{C}\alpha_{k}A_{k})\in {\mathbb{R}}^{\left(B,H,W\right)}$$

where A*_k_* ∈ *ℝ ^B,H,W^* is the k-th feature map.

##### 3.5.1.4 Resize

Interpolate the CAM to the input image size (224x224) to obtain the final class activation map, as defined in Equation 20:

(20) CAMfinal=Interpolate(CAM,size=(224,224),mode='bilinear')∈ℝB,224,224

where the heatmap is resized to the input image size.

##### 3.5.1.5 Normalization

Normalize for visualization, as defined in Equation 21.

(21) $$CAM_{\text{norm}}=\frac{CAM_{final\;}-min(CAM_{final})}{max(CAM_{final\;})-min(CAM_{final})}$$

The normalized CAM_norm_ is imposed upon the input image for visualization purposes.

#### 3.5.2 Grad-CAM++ (gradient-weighted class activation mapping)

Grad-CAM++ is an improved method of Grad-CAM, offering more precise visualization for the predictions made by CNN models (76). It overcomes Grad-CAM’s weaknesses of Grad-CAM regarding the localization of multiple objects and addresses intricate localization processes with the use of higher-order derivatives and pixel-weighting techniques rather than merely activating through gradient averages. As a result, more precise heatmaps with high definition are produced to denote significant areas in an input image with higher clarity and precision, and this significantly helps in visualizing mammographic images for better clinical diagnosis and interpretation.

Let $A^{k}$ ∈ *ℝ ^H×W^* be the k-th feature map from the target layer (e.g., layer4), and y_c_ be the score for class c before softmax. The Grad-CAM++ heatmap is computed as follows:

##### 3.5.2.1 Gradients

Compute the first- and second-order gradients as defined in Equation 22.

(22) $$G_{i,j}^{k}=\frac{\partial y_{c}}{\partial A_{i,j}^{k}},\;G_{i,j,i^{',}j^{\prime }}^{k}=\frac{\partial^{2}y_{c}}{\partial A_{i,j}^{k}\partial A_{i^{',}j^{\prime }}^{k}}$$

##### 3.5.2.2 Pixel-wise weights

Grad-CAM++ relies upon mechanisms involving multiple gradients to assign appropriate weights to every pixel of the feature map to denote its contribution to the prediction of the class, with the assigned weights according to higher-order derivatives as defined in Equation 23.

(23) $$\alpha_{i,j}^{k}=\frac{\frac{\partial^{2}y_{c}}{\partial {\left(A_{i,j}^{k}\right)}^{2}}}{2.\;\frac{\partial^{2}y_{c}}{\partial {\left(A_{i,j}^{k}\right)}^{2}}+\sum i^{\prime }j^{\prime }A_{i^{',}j^{\prime }}^{k}.\;\frac{\partial^{3}y_{c}}{\partial {\left(A_{i,j}^{k}\right)}^{3}}}$$

##### 3.5.2.3 Global weights

Average the pixel-wise weights to get the importance of each feature map as defined in Equation 24:

(24) $$\alpha_{k}=\sum_{i,j}\alpha_{i,j}^{k}$$

##### 3.5.2.4 Heatmap

Combine the weighted feature maps and apply ReLU to obtain the Grad-CAM++ localization map, as defined in Equation 25:

(25) $$L_{\text{Grad}-\text{CAM}++}=\text{ReLU}(\sum_{k}\alpha_{k}A^{k})\in {\mathbb{R}}^{\left(H\times W\right)}$$

##### 3.5.2.5 Resize

Interpolate the heatmap to the input image size (e.g., 224x224) to obtain the final Grad-CAM++ heatmap as defined in Equation 26:

(26) Lfinal=Interpolate(LGrad-CAM++,size=(224,224),mode='bilinear')

##### 3.5.2.6 Normalization

Normalize for visualization as defined in Equation 27.

(27) $$L_{\text{norm}\;}=\frac{L_{final\;}-min(L_{final})}{max(L_{final\;})-min(L_{final})}$$

#### 3.5.3 Score-CAM (score-weighted CAM)

Score-CAM is a gradient-free XAI approach that provides class activation maps to point out the regions of the input image that are most important for the predicted class, such as the lesions in mammograms. Unlike other methods, it assigns importance to the feature maps by the model’s confidence scores, which are less impacted by gradient noise or vanishing gradients (76).

##### 3.5.3.1 Mask generation

Normalize and upsample each feature map *A_k_*, as defined in Equation 28:

(28) $$M^{k}=\text{Interpolate}(\frac{A^{k}-min(A^{k})}{max(A^{k\;})-min(A^{k})},\text{ size}=(224,224))$$

##### 3.5.3.2 Masked image

Apply the mask to the input image I, as represented in Equation 29:

(29) $$I^{k}=I\;.\;M^{k}$$

##### 3.5.3.3 Class score

Pass the masked image through the model to get the score for class c, as represented in Equation 30:

(30) $$s_{c}^{k}=\text{model}{\left(I^{k}\right)}_{c}$$

##### 3.5.3.4 Heatmap

Combine feature maps weighted by scores, as defined in Equation 31:

(31) $$L_{\text{Score}-\text{CAM }}=\text{ ReLU }(\sum_{k=1}^{c}s_{c}^{k}A^{k})$$

The input images are resized and normalized, similar to the methodology in Grad-CAM.

Score-CAM is a highly effective technique in the context of biomedical imaging, particularly for the analysis of mammograms. As it does not involve the concept of gradient information, it eliminates problems of gradient noise or the possibility of vanishing gradients (76).

The ResNet50HierarchicalMultiScale model, as previously described, enhances ResNet50 by incorporating hierarchical and multi-scale attention, allowing layer 4 feature maps to concentrate on critical regions such as lesions in mammograms. The final convolutional layer (layer4) outputs high-level semantic features (2048 channels, typically 7×7 for a 224×224 input), making it well-suited for visualizing class-specific regions through heatmaps.

### 3.6 Training and inference

The model is trained using the Adaptive Moment Estimation (Adam) optimizer, a batch size of 32, and cross-entropy loss for a classification task (8). All experiments were performed on an NVIDIA RTX-A4000 GPU with 16 GB of memory. The number of FLOPs was calculated using a standard profiling library, and the inference time was calculated on the entire test dataset. Although HMC-Net adds more attention modules, which result in more parameters and FLOPs (**Table 2**), the computational cost of training and inference is still manageable. Considering the great improvement in accuracy and interpretability, this is an acceptable compromise for clinical decision support systems. After loading a model for training, a test image is processed, and XAI is utilized to generate a heatmap that visualizes the regions contributing to the predicted class (75).

**Table 2:** Computational complexity and efficiency of ResNet50_HMC compared to baseline models.

Model	Parameters (M)	FLOPs (G)	Training time (h)	Inference time (ms/img)
ResNet50	23	8	2	7
ResNet50_HMC	45	25	3	18

The model is trained using Cross-Entropy Loss, as defined in Equation 32:

(32) $$L=\sum_{c=1}^{\text{num}\_\text{classes}}{\text{t}}_{\text{c}}\text{log}({\text{p}}_{\text{c}})$$

where:

t_c_​: Ground-truth label (1 if class c is correct, 0 otherwise).
p_c_: Predicted probability for class c, computed, as defined in Equation 33:

(33) $${\text{p}}_{\text{c}}=\text{softmax}{\left(\text{Out}\right)}_{c}==\frac{\exp(Out_{c\;)}}{\;\sum_{j=1}^{num\_classes\;}\;\exp(Out_{j\;)}}$$

The loss is minimized using the Adam optimizer as represented in Equation 34:

(34) $$\theta_{t}+1=\theta_{t}-\eta \cdot \nabla_{\theta }L$$

where η =0.0001[8] is the learning rate, and *θ* represents the model parameters.

## 4 Result

### 4.1 Evaluation metrics

To validate the performance of the suggested model in predicting benign and malignant mammograms, multiple metrics have been used, including test accuracy, sensitivity, specificity, and F1-score (59) (4). To evaluate the model’s overall performance throughout the training and testing, the following equations (Equations 35–39) were applied to the test data. In these equations, TP is the true positives, FP is false positives, TN is true negatives, and FN is false negatives. These metrics are calculated as follows:

(35) $$\;\;\text{Accuracy}=\frac{\text{TP }+\text{ TN }}{\text{TN }+\text{ FP }+\text{ FN }+\text{ TP}}$$

(36) $$\text{Sensitivity}=\frac{\text{TP}}{\text{TP}+\text{FN}}$$

(37) $$\text{Specificity}=\frac{\text{TN}}{\text{TN}+\text{FP}}$$

(38) $$\text{Precision}=\frac{\text{TP }}{\text{TP }+\text{ FP}}$$

(39) $$\text{F}1-\text{Score}=\frac{2*\text{Precision}*\text{Sensitivity}}{\text{Precision}+\text{Sensitivity}}$$

### 4.2 Performance analysis of the proposed ResNet50 hierarchical multi-scale cross attention network

**Figure 5:**
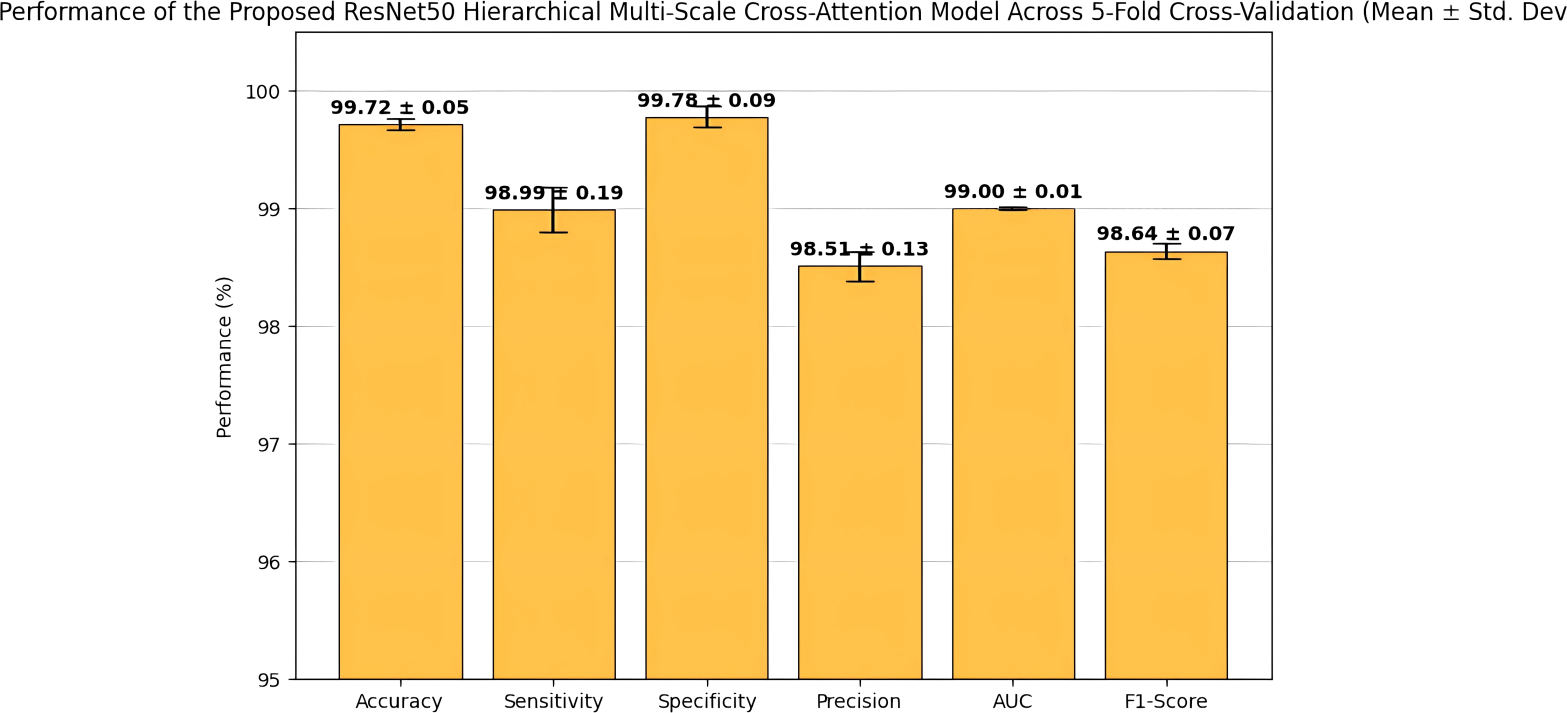
Error bars showing the performance of the ResNet50-HMC model across five-fold cross-validation.

**Figure 6:**
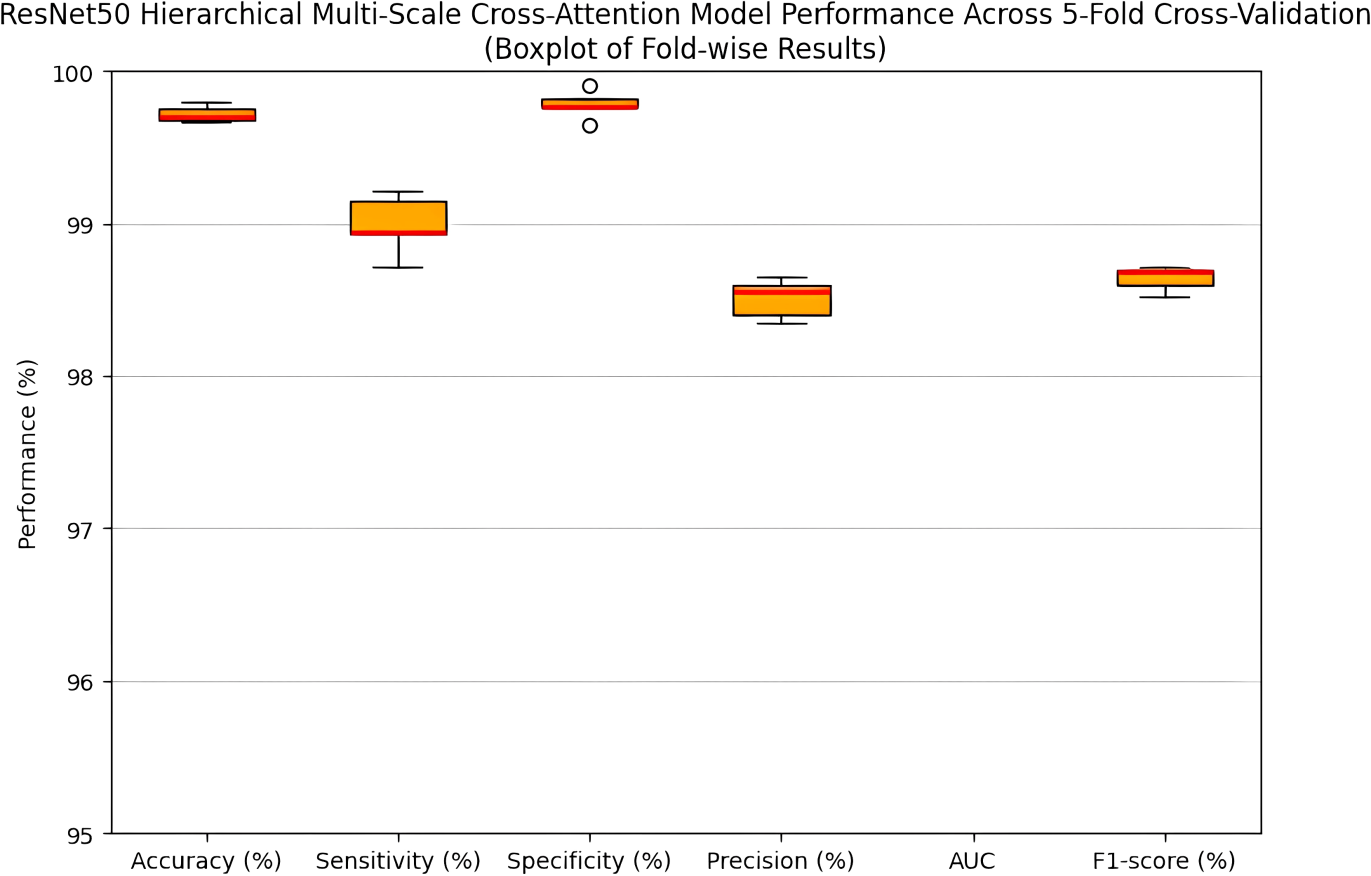
Boxplot showing the performance of the ResNet50-HMC model across five-fold cross-validation.

The performance of the proposed ResNet50HierarchicalMultiScale model and its variants in mammogram classification for the MIAS dataset was evaluated using 5-fold cross-validation. The key performance metrics of accuracy, precision, recall, F1-score, AUC-ROC, and specificity are reported as follows: **Table 3**: Performance (mean ± standard deviation) of the proposed ResNet50-HMC model obtained by 5-fold cross-validation. The performance has been consistently high with respect to all key metrics. Low standard deviation values signify the robustness and reliability of the model for classifying mammograms. **Figures 5** and **6** illustrate the performance variations of the ResNet50-HMC model in five folds with mean and SD for different evaluation metrics. **Table 4**: Performance comparison-mean of various metrics in 5-fold cross-validation for four different configurations proposed ResNet50HierarchicalMultiScale (proposed with hierarchical self-attention modules HA1, HA2, and multi-scale cross-attention modules CA23, CA34), ResNet50Hierarchical (only hierarchical self-attention), ResNet50Multi-Scale (only multi-scale cross-attention), and Baseline ResNet50 (no attention). The performances obtained from the proposed model, as evident from **Table 4**, were superior with a mean accuracy of 0.9972 with a low variance-standard deviation of 0.05, which indicates the robust generalization. A high precision, recall, F1-score, and AUC-ROC indicate the ability of this model in class discrimination with elevated specificity, highlighting its superiority in correctly identifying the normal cases, which is most crucial for reducing the false positivity rates in clinical settings.

**Table 3:** Performance of proposed ResNet50-HMC model (mean ± std. dev.) with 5-fold cross-validation.

Metric	Fold 1	Fold 2	Fold 3	Fold 4	Fold 5	Average ± std dev
Accuracy (%)	99.68	99.75	99.70	99.80	99.67	99.72%± 0.05
Sensitivity (%)	98.72	99.21	98.93	99.15	98.94	98.99± 0.19
Specificity (%)	99.65	99.82	99.91	99.76	99.76	99.78%± 0.09
Precision (%)	98.40	98.65	98.55	98.60	98.35	98.51%± 0.13
AUC	.99	.98	.99	.98	.99	.99± 0.01
F1-score (%)	98.52	98.71	98.60	98.69	98.68	98.64%± 0.07

**Table 4:** Performance comparisons of the proposed ResNet50 hierarchical multi-scale cross attention network model variants on MIAS mammograms across five-fold cross-validation.

Model variant	Accuracy	Precision	Sensitivity	Specificity	F1-score	AUC-ROC
ResNet50_HMC	99.72%	98.51%	98.99%	99.78%	98.64%	.99
No Multi-Scale Attention/ResNet50Hierarchical	99.53%	98.33%	98.91%	99.67%	98.59%	.99
No Hierarchical Attention/ResNet50Multi-Scale	99.41%	98.23%	98.90%	99.56%	98.54%	.99
Baseline ResNet50	99.24%	98.13%	98.86%	99.45%	98.49%	.99

**Table 5** shows the results of the mean of the metrics for five different model conditions: the proposed model ResNet50HierarchicalMultiScale, the baseline model ResNet50, VGG19, VGG16, and Vision Transformer (ViT) models. From **Table 4**, the performance of the proposed model is the best with an accuracy of 0.9972 (± 0.05), precision of 0.9851 (± 0.13), recall of 98.99 (± 0.19), F1 Score of 0.9864 (± 0.07), AUC-ROC of 0.99 (± 0.01), and specificity of 0.9978 (± 0.09). These results, with low standard deviations, tend to reflect robust generalization and better discriminative powers compared to the baseline models. Of particular importance is the high specificity that is essential for minimizing false positives in the clinical setting, which, along with a near-perfect AUC-ROC, speaks of the model’s effective separation between classes.

**Table 5:** Classification performance of models on MIAS mammogram dataset for tumor classification across five-fold cross-validation.

Model	Class	Accuracy	Precision	Recall	Specificity	F1-score	AUC-ROC
ResNet50_HMC	3-class	99.72	98.51	98.99	99.78	98.64	.99
ResNet50	3-class	99.24	98.13	98.86	99.45	98.49	.99
VGG19	3-class	97.8	92.01	95.8	96.9	93.81	.97
VGG16	3-class	98.53	97.04	97.62	98.5	98.02	.99
ViT	3-class	84.1	83.31	83.5	83.5	83.4	.835

**Figure 7:**
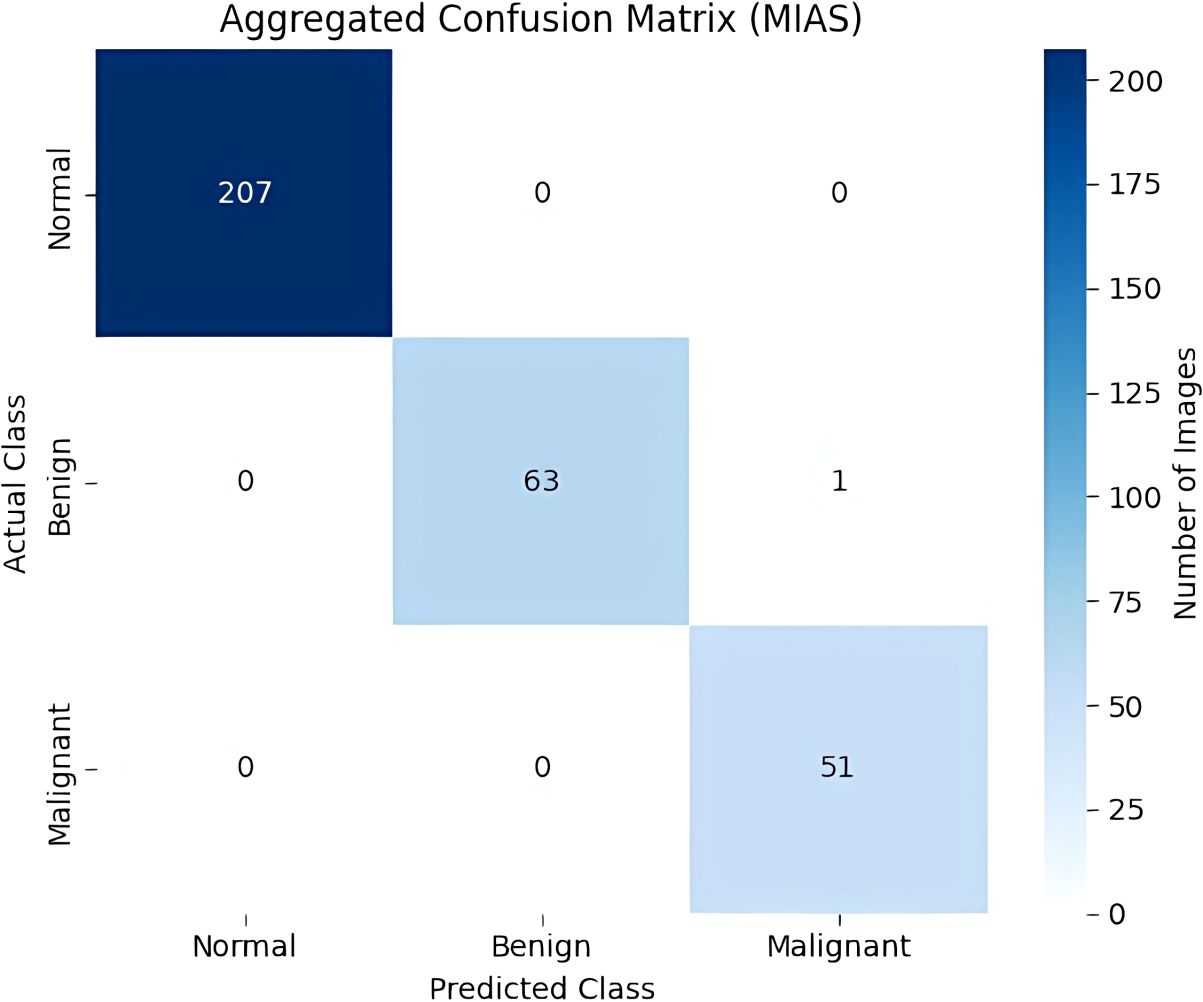
Aggregated confusion matrix.

The aggregated confusion matrix (**Figure 7**) reveals near-perfect performance. All 207 normal and 51 malignant cases were correctly classified, with only one benign case incorrectly classified as malignant. Most importantly, no malignant cases were false negatives, ensuring zero false negatives for the highly important malignant class. The remaining 0.28% error is insignificant and does not affect diagnostic safety, ensuring a negligible probability of a missed cancer diagnosis while retaining extremely high accuracy.

### 4.3 Training and validation performance of ResNet50 hierarchical multiscale cross attention network for mammogram classification

**Figure 8:**
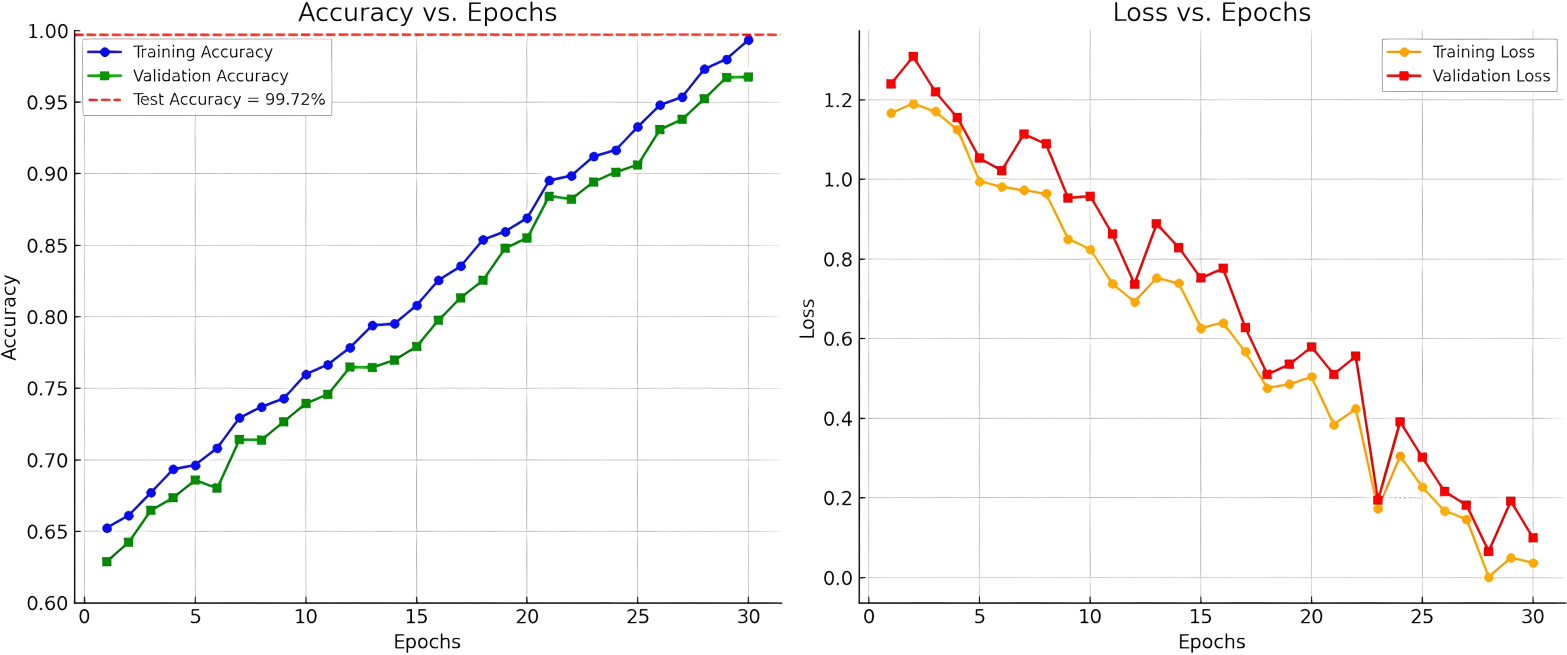
Classification performance curves (accuracy and loss) of ResNet50-hierarchical multi-scale model on MIAS dataset.

To demonstrate learning dynamics and resilience of our proposed ResNet50Hierarchical Multi-Scale model in tumor classification on mammograms in the MIAS dataset, we provide two graphs in **Figure 8**: (a) Accuracy *vs*. Epochs and (b) Loss *vs*. Epochs. These graphs demonstrate training and validation accuracy over training epochs, with the test accuracy of 0.9972, obtained after training and validation, represented as a dashed red line in the Accuracy *vs*. Epochs graph. The Accuracy *vs*. Epochs (left) graph tends to demonstrate a gradual increment in training accuracy with minor fluctuations, thereby indicating consistent learning of distinguishing features in our model through hierarchical attentions (HA1, HA2) and cross-attention (CA23, CA34) for capturing characteristics on multiple scales. The dashed red line with an accuracy of 0.9972 on the Accuracy *vs*. Epochs graph clearly justifies good tumor classification on normal, benign, and malignant samples. The Loss *vs*. Epochs graph indicates smooth convergence with steadily reducing loss and almost parallel validation loss curves. This has been achieved with Adam with a learning rate of 0.0001 and Cross-Entropy Loss.

### 4.4 Interpretability through XAI visualizations

**Figure 9:**
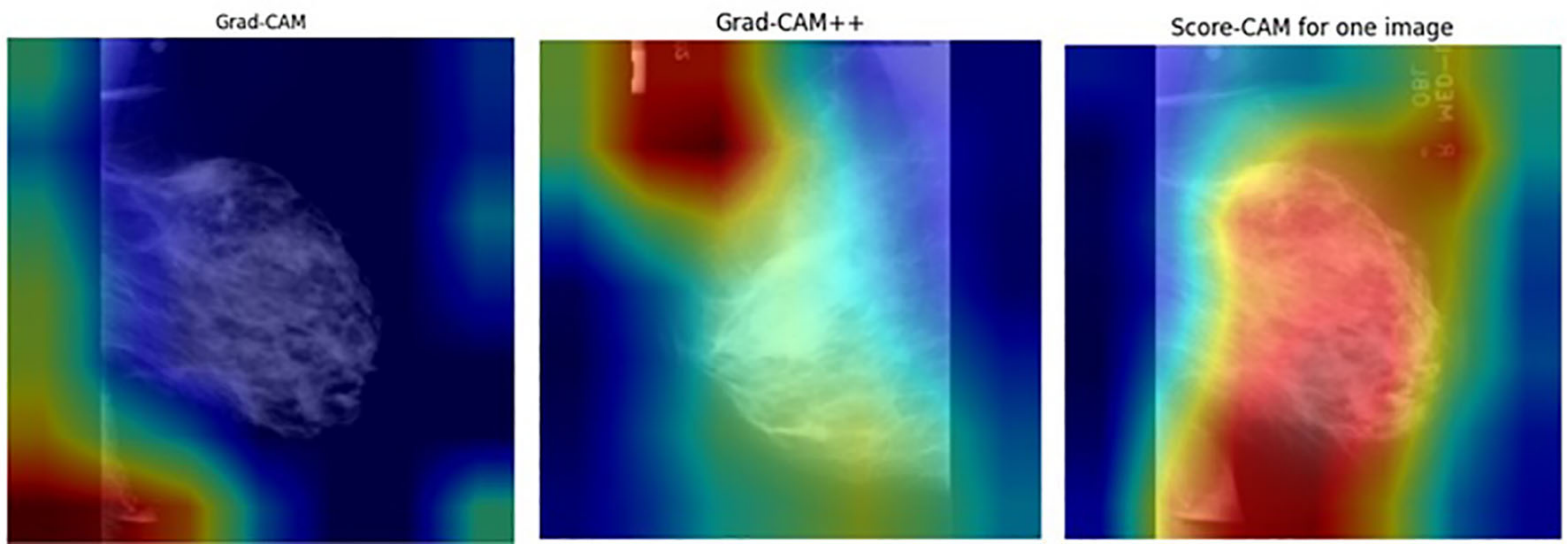
Heatmap visualization for benign mammograms.

**Figure 10:**
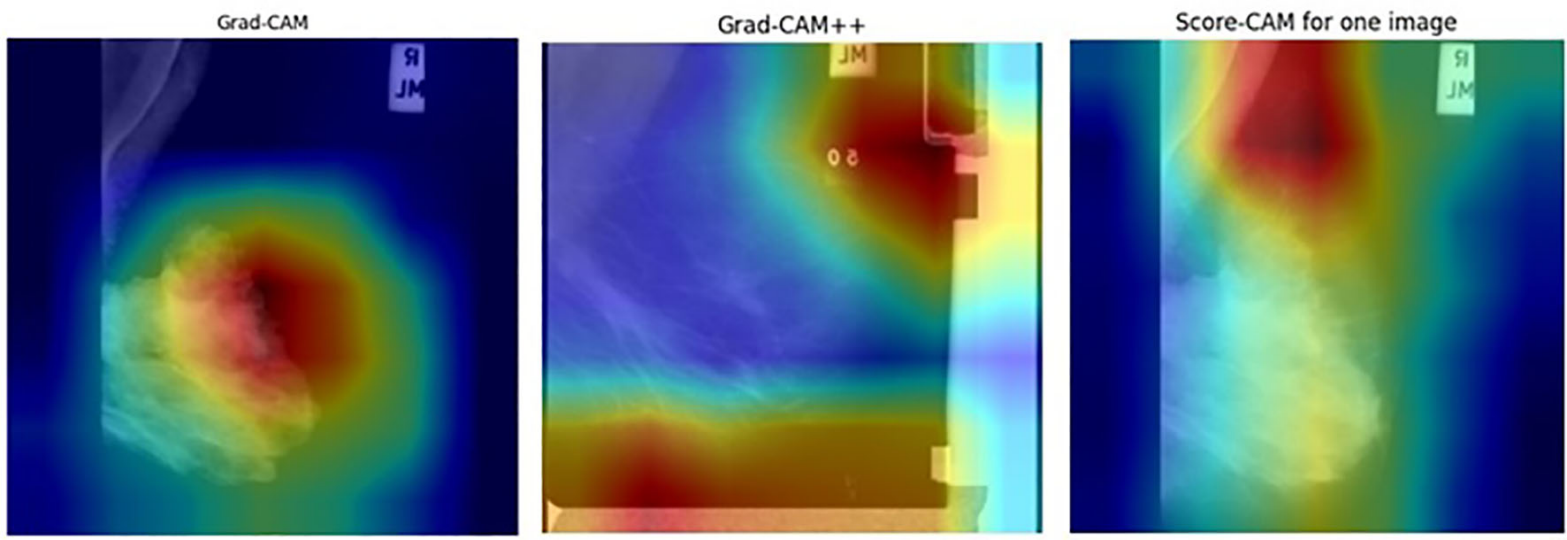
Heatmap visualization for malignant lesion mammograms.

For this research, Grad-CAM, Grad-CAM++, and Score-CAM techniques were used to emphasize areas that indicate decisions made by a diagnostic model. **Figures 9** and **10** conclusively demonstrate the produced heat maps, which identify areas in an image that are most crucial for classification. The red and yellow coloration in these heat maps identifies areas of close attention by the model, which marks their relevance in mammogram diagnostics, whereas other areas are illustrated in cooler colors, which are less significant areas of consideration (75). The capability of the proposed model to generate meaningful and transparent heat maps using Grad-CAM, Grad-CAM++, and Score-CAM techniques further establishes that the proposed model concentrates on significant areas of diagnostics, making it highly efficient in medical diagnostics as well.

#### 4.4.1 Comparison in the context of RESNET50 MULTISCALE HIERARCHICAL

The ResNet50MultiScaleHierarchical model improves ResNet50 with hierarchical and multi-scale cross-attention, making its feature maps (for example, those at layer 4) concentrate on informative locations in an image (for example, lesions in mammograms). The choice of CAM technique affects the interpretability of these feature maps:

##### 4.4.1.1 Grad-CAM

Establishes a baseline technique for visualization. It works well for a single object in terms of localization, but cannot capture fine details contained in a mammogram with multiple regions of interest.

##### 4.4.1.2 Grad-CAM++

Utilizes higher-order gradients to make detailed heatmaps, which are required for complex images such as medical images, where multiple lesions or patterns must be emphasized.

##### 4.4.1.3 Score-CAM

It does not rely on gradients, hence ensuring robustness, and is particularly helpful when there is an attention mechanism because the gradients are noisy. Its computation is intensive.

Grad-CAM++ performs well in mammogram classification by giving precise localization of lesions, whereas Score-CAM provides robust and reliable visualizations even when one is dealing with complex attention-based features. Attention mechanisms within the model further refine feature maps to provide heatmaps, highlighting clinically important regions and improving interpretability for medical diagnosis.

For the quantitative validation of the explanations, the heatmaps were normalized to the range [0,1] and binarized with a threshold of 0.5. The MIAS ROI masks were resized to have the same resolution as the heatmap, and the IoU was calculated against the annotations. The average IoU per method was calculated over all test samples in 5-fold cross-validation. **Table 6** shows the IoU values, which clearly show the alignment of the highlighted areas with the lesions.

**Table 6:** Quantitative assessment of XAI methods using Intersection over Union (IoU).

XAI method	Threshold	Average IoU	Description
Grad-CAM	0.5	0.62	Most salient regions captured well
Grad-CAM++	0.5	0.65	Slightly better localization
Score-CAM	0.5	0.68	Produces smoother heatmaps

Grad-CAM++: enhanced Grad-CAM using higher-order gradients.

### 4.5 Comparative performance analysis against state-of-the-art methods

The performance of the proposed ResNet50HierarchicalMultiScale model is compared with the state-of-the-art methods. A comparison is made with existing prior approaches in **Table 7**.

**Table 7:** A comparative performance assessment between the proposed model and existing state-of-the-art techniques.

Study	Technique	Breast dataset	Accuracy (%)
Naveed Chouhan et al. (77)	Hybrid (DFeBCD) + SVM)	IRMA mammogram	80.50
Madallah Alruwaili et al. (78)	MOD-RES	MIAS	89.50
Steven Squires et al. (79)	Ensemble (ResNet18 + DensNet161)	Mammogram	84.01
Sreelekshmi et al. (80)	Depth-wise separable convolution and Swin transformer	IDC	98.32
Spanhol et al. (81)	AlexNet	BreakHis	85
Sanyal et al. (82)	Hybrid Ensemble of Deep Convolution Features	BACH	95
Han et al. (83)	Structured Deep Learning	BreakHis	95
Jiamei Sun et al. (84)	ResNet50, CaffeNet and GoogleNet	BreakHis	95
Kulkarni et al. (85)	ResNet 152 and fully connected layer	IDC	91
Selina et al. (86)	ResNet50 V2 and light boosting classifier	IDC	95
Payal et al. (87)	Various DL models	BACH	97.50
Dalal Bardou et al. (88)	Ensemble model	BreakHIs	97
Soumya et al. (89)	ML classifier CatBoost	IDC	92.55
Tummala et al. (58)	Ensemble of SwinTs	BreakHis	96.0
Zeynali et al. (8)	Hybid CNN transformer with Xception Feature Fusion	BreaKHis	99.62
Gupta V et al. (90)	Modified residual networks	BreaKHis	99.5
B Dalal et al. (88)	CNN-SVM	BreaKHis	86.3
Jiang Y et al. (91)	Small SE-ResNet	BreaKHis	93.7
Hameed Z et al. (92)	Ensemble of VGG16 and VGG 19	WSI	95.3
Proposed Approach	ResNet50_HMC	MIAS	99.72

### 4.6 Statistical analysis

For statistical validation of performance comparisons among the four classification models, ResNet50, ResNet50 with Hierarchical Attention, ResNet50 with Multi-Scale Attention, and the proposed ResNet50 with Hierarchical Multi-Scale Cross Attention (ResNet50_HMC), a Friedman test along with the Nemenyi *post-hoc* test (93) was performed, which involved accuracy measures obtained by running a 5 fold cross-validation experiment on the accuracy achieved by each model on the MIAS mammography image data set. From the Friedman test, a statistical value of 14.755 was obtained, and a p-value of 0.002, which is less than the significance level set at 0.05, confirming that a statistically significant difference exists among the performances of the four models. Upon performing a *Post-hoc* Nemenyi test, it was determined that a statistically significant improvement was obtained by the proposed ResNet50_HMC method (average accuracy = 0.9972 ± 0.05) over ResNet50, but that there existed no inter-model significant difference among Hierarchical Attention, Multi-Scale Attention, and HMC Attention, as specified in **Table 8**, which implies that although each mechanism contributes significantly to accuracy, it is indeed the synergy that leads to an extreme level of accuracy by HMC design. The matrix comparing all pairs of methods via a *post-hoc* Nemenyi test is illustrated in **Table 9**.

**Table 8:** Nemenyi *post-hoc* test (p-values).

Model variant	ResNet50	ResNet50_Hierarchical	ResNet50_MultiScale	ResNet50_HMC
ResNet50	1.0000	0.0496	0.6111	0.0022
ResNet50_Hierarchical	0.0496	1.0000	0.5327	0.7610
ResNet50_MultiScale	0.6111	0.5327	1.0000	0.0919
ResNet50_HMC	0.0022	0.7610	0.0919	1.0000

**Table 9:** Nemenyi *post-hoc* analysis (pairwise comparisons).

Comparison	P-value	Interpretation
ResNet50 *vs* ResNet50_HMC	0.002	Significant difference (HMC performs better)
ResNet50 *vs* ResNet50_Hierarchical	0.049	Borderline significance (slight improvement)
ResNet50 *vs* ResNet50_MultiScale	0.611	Not statistically significant
ResNet50_HMC *vs* ResNet50_Hierarchical	0.761	Not statistically significant
ResNet50_HMC *vs* ResNet50_MultiScale	0.091	Not statistically significant
ResNet50_Hierarchical *vs* ResNet50_MultiScale	0.532	Not statistically significant

**Figure 11:**
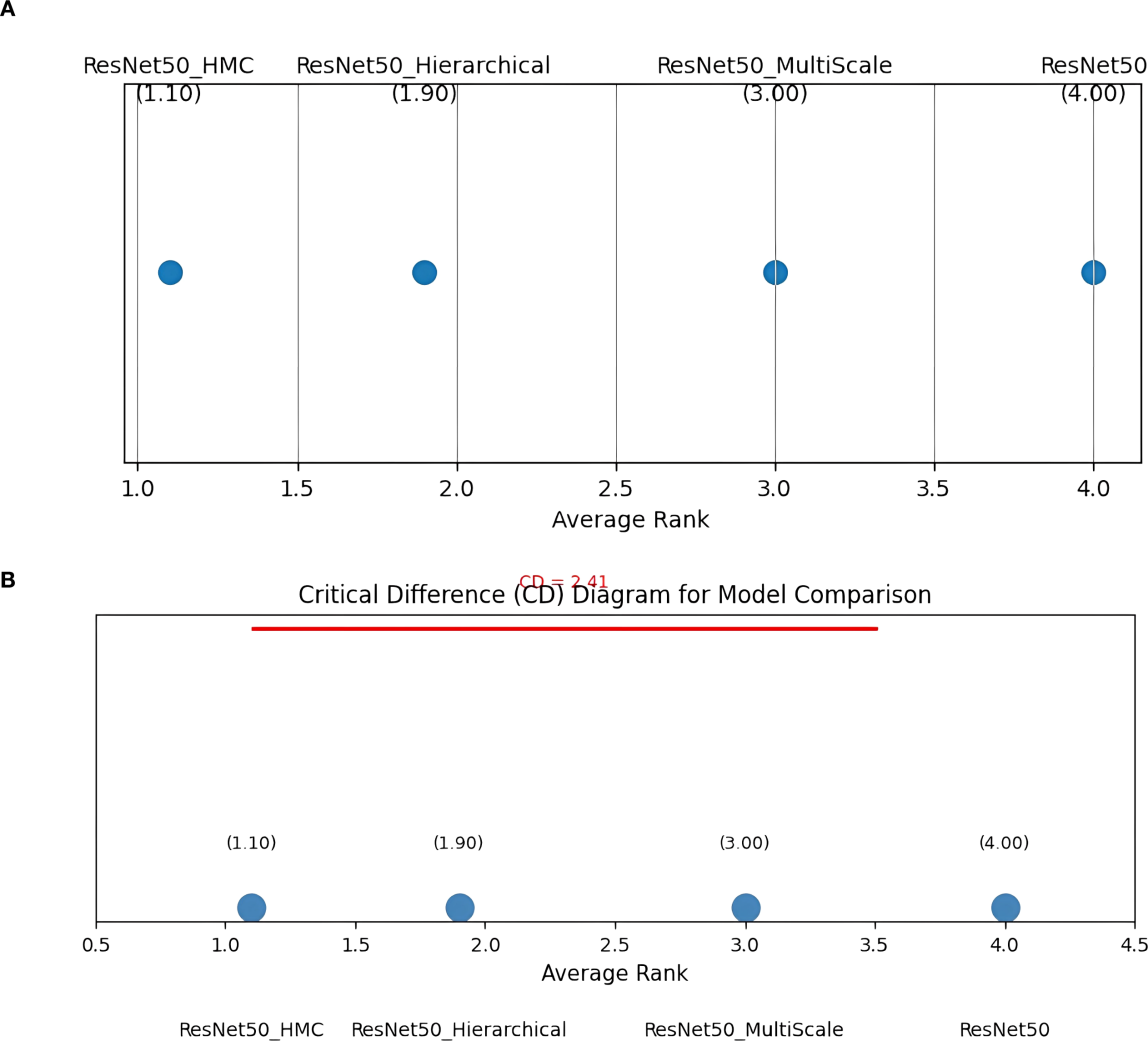
**(a)** Average rank of models. **(b)** Critical difference (CD = 2.41) diagram for statistical comparison.

As an additional validation step, a Critical Difference (CD) diagram was constructed, presented in **Figure 11a, b**, which reflects the average ranks of the evaluated models (lower rank indicates better performance) based on their accuracy across folds. This visualization provides a clear comparison of their relative performance and highlights the statistical distinctions identified through the Friedman and Nemenyi analyses. In this diagram, models are ranked from best to worst, with a horizontal CD line indicating whether differences are statistically significant at the 95% confidence level. Models connected by the CD line are not significantly different, whereas unconnected models indicate a meaningful statistical difference. The CD value, computed as 2.41 for 4 models, 5 folds, and a significance level of α = 0.05 using the formula defined in Equation 40:

(40) $$CD=q_{\text{α}}\sqrt{\frac{k\left(k+1\right)}{6N}}$$

where q_α_ = 2.949 (Nemenyi critical value), k = 4 (models), and N = 5 (folds), is depicted as a horizontal line as shown in **Figure 10b**. The diagram confirms that ResNet50 with HMC-Attention (average rank: 1.10) is significantly better than ResNet50 (average rank: 4.0), with Hierarchical (rank: 1.90) and Multi-Scale (rank: 3.0) showing no significant differences among themselves.

The Friedman and Nemenyi tests, along with the CD diagram, show that ResNet50 with HMC-Attention (rank 1.10) significantly outperforms the standard ResNet50 baseline (rank 4.0), with a rank difference (2.9) exceeding the critical difference (2.41) and a corresponding large effect size (Cohen’s d ≈ 1.2). Hierarchical (1.90) and Multi-Scale (3.0) variants do not differ significantly among themselves. These results not only provide robust statistical evidence of the effectiveness of ResNet50_HMC but also suggest potential clinical relevance, as improved model performance could enhance diagnostic accuracy and decision support in mammography.

## 5 Conclusion and future work

This research introduced a ResNet50-based framework for mammogram classification, enhanced with hierarchical self-attention (HA1, HA2), multi-scale cross-attention (CA23, CA34), and explainable AI (Grad-CAM, Grad-CAM++, Score-CAM). By addressing both intra- and inter-layer dependencies, the network presents an effective combination of local enhancements and global semantic knowledge, centered on relevant regions to the diagnostics. On the MIAS test images, it demonstrated greater efficacy, reaching mean accuracy at 0.9972 (± 0.05%), precision of 0.9851(± 0.13%), recall of 0.9899(± 0.19%), F1-score of 0.9864(± 0.07%), AUC-ROC value of 0.9990(± 0.05%), and specificity of 0.9978(± 0.09%) outperforming other models like ResNet50, VGG16, and VGG19. Statistical validation, as determined by the Friedman and Nemenyi tests, confirmed significant improvements over baseline models, highlighting the synergistic effect of integrating hierarchical and multi-scale attention. XAI visualizations subsequently validated the model’s focus on critical areas, strengthening trust and interpretability in clinical diagnostics. While Score-CAM is computationally costly, its robustness complements the fine localization of Grad-CAM++, providing a comprehensive interpretability framework. Overall, the proposed ResNet50HierarchicalMultiScale model demonstrates strong potential as an accurate and interpretable framework for mammogram classification. Future research could explore optimizing the computational efficiency of Score-CAM, integrating additional modalities (e.g., ultrasound), and validating the proposed framework on larger full-field digital mammography (FFDM) datasets to further strengthen its generalizability. The ResNet50HierarchicalMultiScale model, with its high accuracy, robust statistical validation, and interpretable visualizations, exhibited a substantial improvement in automated mammogram analysis, providing a reliable and transparent tool for clinical decision support in breast cancer diagnostics.

## Data Availability

Publicly available datasets were analyzed in this study. This data can be found here: J. Suckling, “The mammographic images analysis society digital mammogram database,” in Exerpta Medica. International Congress Series, 1994, 1994, vol. 1069, pp. 375–378.
